# Pharmacotherapy for Nocturia

**DOI:** 10.1007/s11934-018-0750-y

**Published:** 2018-02-09

**Authors:** Karl-Erik Andersson, Philip Van Kerrebroeck

**Affiliations:** 1 Lund University, Lund, Sweden; 2 Institute for Regenerative Medicine, Wake Forest University School of Medicine, Winston Salem, NC USA; 3 Institute of Clinical Medicine, Department of Obstetrics and Gynecology, Aarhus University, Palle Juul-Jensens Boulevard 99, 8200 Aarhus N, DK Denmark; 4 Department of Urology, Maastricht University Medical Center, Maastricht, The Netherlands

**Keywords:** Global polyuria, Nocturnal polyuria, Reduced bladder capacity, Overactive bladder, Pharmacological principles

## Abstract

**Purpose of Review:**

To assess current pharmacological principles used for treatment of nocturia/nocturnal polyuria.

**Recent Findings:**

The pathophysiology of nocturia is often multifactorial, but two main mechanisms have been identified, occurring alone or in combination: low functional bladder capacity and nocturnal polyuria. The multifactorial pathophysiology not only implies several possible targets for therapeutic intervention but also means that it is unlikely that one treatment modality including drugs will be successful in all patients. Drugs approved for the treatment of male LUTS and male and female OAB are known to be far more effective for treatment of the daytime symptoms than for nocturia.

**Summary:**

Several pharmacological principles have been tested with varying success. The treatment of choice should depend upon the main underlying cause, thus aiming primarily to increase bladder capacity by counteracting detrusor overactivity and/or reducing nocturnal polyuria. Using current available agents, effective, personalized treatment should be designed taking into account gender, co-morbidities, and identified etiological factors. However, there is a medical need for new, approved drugs for treatments for patients with nocturia.

## Introduction

The etiology of nocturia is multifactorial [1, 2]. The most frequent cause is nocturnal polyuria, that can be defined as night-time urine output higher than 20% of total daily urine output for younger adults and higher than 33% for older adults. Causative factors can be grouped into five main categories: global polyuria, nocturnal polyuria, reduced bladder capacity, sleep disorders, and circadian clock disorders [1]. However, many patients will have more than one factor involved. Increased urine production can be caused by the reabsorption of fluid during sleep, solute-related diuresis, diuretic medications, and the ingestion of excess fluid in the evening. Diminished functional bladder capacity can be due to OAB or partial bladder outlet obstruction resulting in increased residual urine volume. Treatment approaches not only have to consider these factors but also to take into account gender, co-morbidities and identified etiological factors.

## Drug Alternatives

Nocturia and its treatment have been the subject of a number of reviews focusing on both men [3, 4••] and women [5]. The multifactorial pathophysiology not only offers several possible targets for therapeutic intervention but also means that it is unlikely that one treatment modality including drugs will be successful in all patients. Many pharmacological principles have been tested with varying success. Currently, available therapeutic modalities either have modest efficacy or are targeted at subpopulations of the entire group of patients with nocturia [6]. Drugs approved for the treatment of nocturia associated with benign prostatic obstruction (BPO) and OAB are known to be far more effective for treatment of the daytime lower urinary tract symptoms (LUTS) of frequency, urgency, straining, weak stream and stress, and urgency incontinence than for nocturia, and there is a medical need for new, effective, and approved drug treatments.

### Vasopressin V2 Receptor Agonists

Desmopressin (DDAVP) has for a long time been in clinical use for the treatment of nocturnal polyuria, and there are several recent reviews of the mechanism of action and clinical use of the drug [4••, 7–10]. DDAVP is a synthetic analogue of antidiuretic hormone (ADH) and it binds to V2 receptors in the renal collecting duct and stimulates water reabsorption. DDAVP is available in formulations for oral, parenteral, and nasal administration. Because of symptomatic hyponatremia with water intoxication, which occurred after intranasal or intravenous administration of desmopressin, the U.S. Food and Drug Administration (FDA) and the European Medicines Agency (EMA) removed the indication for the treatment of primary nocturnal enuresis from all intranasal preparations of desmopressin in 2007. However, in 2017, FDA approved desmopressin nasal spray (Noctiva^R^, desmopressin acetate) for the “treatment of nocturia due to nocturnal polyuria in adults who awaken at least 2 times per night to void.” An oral desmopressin lyophilisate formulation (Nocdurna^R^) requiring no concomitant fluid intake is currently the most widely used DDAVP preparation.

DDAVP has shown efficacy in nocturia due to nocturnal polyuria as evident from numerous reviews [4••, 7–12]. Weiss et al. [13] performed a 4-week, randomized, double-blind study comparing 10, 25, 50, or 100 μg desmopressin (oral dispersible desmopressin (Minirin^R^ Melt) versus placebo in adults with defined nocturia. The study included 757 patients reporting three or more nocturic episodes per night with 90% due to nocturnal polyuria. Increasing doses of desmopressin were associated with decreasing numbers of nocturnal voids and voided volume, greater proportions of subjects with > 33% reduction in nocturnal voids, and increased duration of first sleep period. Post hoc analyses by gender suggested a lower minimum effective dose for women. Based on their results, the authors recommended lower and gender-specific dosing to reduce the small but clinically significant risk of hyponatremia.

Women appear to be more sensitive to desmopressin than men. This has been attributed to the fact that the gene for the vasopressin V2 receptor is located on the X chromosome in a region with high probability of escape from inactivation; this may lead to phenotypic sex differences, with females expressing higher levels of transcript than males [14, 15]. The lowest therapeutically beneficial dose of desmopressin (orally disintegrating tablet) has been determined as 50 μg for men [16] and 25 μg for women [17].

Hyponatremia (defined as serum sodium < 130 mmol/L) is the main risk associated with desmopressin therapy [18]. It was found in 4.9% of all patients in high-dose desmopressin tablet studies. Patients most likely to experience desmopressin-induced hyponatremia were older (nearly all > 65 years), had lower body mass, higher urine output, lower basal serum sodium level, and lower creatinine clearance at baseline than those who did not develop hyponatremia. Choi et al. [19] retrospectively analyzed data from 172 patients who were prescribed desmopressin for nocturnal polyuria at a urology clinic from September 2010 through February 2013. Hyponatremia (< 135 mmol/l) was found in 24 patients (14%), and it was severe in 7 (< 126 mmol/l). They concluded that patients with advanced age (≥ 65 years) and low hemoglobin are at risk of desmopressin-associated hyponatremia.

To explore risk factors for desmopressin-induced hyponatremia and evaluate the impact of a serum sodium monitoring plan, Juul et al. [20] performed a meta-analysis of data from three clinical trials of desmopressin in nocturia. Patients received placebo or desmopressin orally disintegrating tablet (ODT). Juul et al. [20] showed that the incidence of hyponatremia can be reduced by using minimum effective gender-specific dosing with the ODT formulation of desmopressin (25 μg in women, 50 μg in men). In patients with nocturia, treated with low-dose desmopressin combined with a sodium monitoring plan during the first month of treatment, mainly mild, non-clinically significant hyponatremia was observed.

Desmopressin is the only therapeutic agent to be highly recommended for treating nocturnal polyuria by the International Consultation on Incontinence (ICI) committee [10].

Fedovapagon (VA106483), a novel non-peptide drug, is a selective V2 receptor agonist, currently in phase II/III development. Preliminary data showed that the antidiuretic effect of fedovapagon can be controlled by dose, giving the potential to improve the risk/benefit profile of V2 receptor agonists in the treatment of nocturia [21].

### Diuretics

The proposed rationale for using diuretics in the treatment in nocturia has been to reduce salt and water load in the body prior to bedtime, and positive effects have been documented with, e.g., bumetanide [22] and furosemide [23].

The efficacy of 1 mg bumetanide was evaluated in an RCT of 28 patients (15 men) in general practice [22]. During the placebo period, the weekly number of nocturia episodes was 13.8 and during bumetanide treatment the number was reduced by 3.8. Ten men with BPE did not improve with bumetanide.

A randomized controlled trial (RCT) of 49 men with nocturnal polyuria evaluated furosemide 40 mg given 6 h before sleep [23]. In the 43 men completing the study, there was a significant reduction in night-time frequency (−0.5 and 0) and percentage night-time voided volume (−18 and 0%) in those taking frusemide compared with placebo. Of patients on active treatment, seven of 19 had a reduction in night-time frequency of ≥ 1, compared with only one of 20 on placebo, and 14 of 21 felt that frusemide had helped their nocturia, compared with only five of 22 on placebo. Combined furosemide and desmopressin was shown to be an effective and well-tolerated treatment for nocturia in the elderly [24](see Combinations).

### Nonsteroidal Anti-inflammatory Drugs

One of the rationales for using nonsteroidal anti-inflammatory drugs (NSAIDs) in the treatment of nocturia is that prostanoids may be involved in the pathophysiology of OAB [25], and the fact that several NSAIDs have shown efficacy in the treatment of this condition [10]. The effects of several NSAIDs, e.g., diclofenac [26] and celecoxib [27] have been evaluated specifically in patients with nocturia.

In a randomized crossover study on 26 patients with nocturnal polyuria (male *n* = 20; female *n* = 6), Addla et al. [26] studied the effects of diclofenac (50 mg daily, taken in the evening). The mean nocturnal frequency significantly decreased from 2.7 to 2.3 and the mean ratio of night-time to 24-h urine volume decreased from 44 to 39%. No significant side effects were reported. A prospective, randomized, double-blind, placebo-controlled study of celecoxib 100 mg versus placebo was performed in 80 men with BPE and 2 nocturia/night [27]. In the celecoxib group, mean nocturnal frequency decreased from 5.2 to 2.5 compared with 5.3 to 5.1. No significant side effects were reported in either study. In some studies, NSAIDs have been combined with various treatment modalities [e.g., 28] (see Combinations).

### Selective α_1_-Adrenoceptor Antagonists

The main rationale for treatment of nocturia with selective α_1_-adrenoceptor (AR) antagonists is that the drugs have a documented effect in the treatment of LUTS, including nocturia [29]. However, the precise mechanism behind their effect on nocturia is yet to be elucidated. α_1_-ARs are distributed both in the LUT and structures in the CNS relevant for micturition control [30], and α_1_-AR antagonists have been shown to reduce activity in afferent pathways of micturition [31], which may explain effects on urinary voiding frequency. Kim et al. [32] found that in patients who had a low prostatic urethra angle, nocturia improved by administration of α_1_-AR antagonist monotherapy, and suggested that the prostatic urethra angle could be a predictor of response to these drugs. However, why α_1_-AR antagonists should reduce nocturnal polyuria is unclear.

Kim et al. [31] investigated improvement in nocturia and nocturnal polyuria after silodosin administration by using a 3-day frequency–volume chart in a prospective multicenter study and found a significant reduction of nocturnal urine volume at 12 weeks compared to screening (*p* = 0.001). Roehrborn et al. [29] not only hypothesized that reduction of nocturnal polyuria combined with improved functional bladder capacity are potential mechanisms of action of α_1_-AR antagonists on nocturia, but also that this effect it is related to α_1_-AR subtype selectivity as none of the individual α_1_-AR antagonists without subtype selectivity has consistently shown a significant reduction in nocturnal voiding episodes [33]. A multicenter 12-wk trial randomized men to receive silodosin, tamsulosin, or placebo and found that only silodosin significantly reduced nocturia versus placebo (*p* = 0.013) and the change from baseline was − 0.9, − 0.8, and − 0.7 for silodosin, tamsulosin, and placebo, respectively [34]. Several studies on the effect on nocturia have used α_1_-AR blockade as comparator arms or in combination with other drugs [4].

The effects of α_1_-AR antagonists on nocturia in females do not seem to have been widely studied. Kim et al. [35] investigated 296 women with women with LUTS (Qmax ≤ 15 mL/s, International Prostate Symptom Score (IPSS) ≥ 8) including nocturia (void/night ≥ 1) nocturia, who completed voiding diary, a questionnaire on the Medical Outcomes Study (MOS) sleep scale, and underwent follow-up evaluation after 4 weeks of treatment (tamsulosin, 0.2 mg, once daily). Clinical parameters, including the IPSS, the bother score, the Qmax, and the PVR, improved significantly from baseline after treatment, and the change in nocturia was − 1.12. The authors concluded that tamsulosin significantly improved nocturia and sleep quality as well as LUTS in women with low Qmax.

Several studies [36] have suggested that combined antimuscarinic + α_1_-AR antagonist treatment is more effective than monotherapy or placebo in men with OAB symptoms, including nocturnal frequency episodes. However, such an effect could not always be demonstrated. For example, Chapple et al. [37] in a randomized controlled trial comparing a group receiving placebo plus α_1_-AR antagonist treatment with a tolterodine ER plus α_1_-AR antagonist group found significantly greater reductions in nocturnal urgency episodes (0.5 vs 0.3; *p* = 0.0378) with the combination, but not on nocturnal frequency.

### 5α-Reductase Inhibitors (Alone or in Combination)

Nocturia improvement in men with BPO treated medically has been modest. The Department of Veterans Affairs Cooperative Study Trial that looked at a cohort of 1078 men with BPH given either terazosin, finasteride, a combination of the two, or a placebo found episodes of nocturia decreased from 2.5 to 1.8, 2.1, 2.0, and 2.1 average episodes, respectively. No drug was found to be dramatically better at treating nocturia than the placebo [38]. A post-hoc subgroup analysis of self-reported nocturia in the Medical Therapy of Prostatic Symptoms trial of men with LUTS found mean nocturia was reduced by − 0.35, − 0.40, − 0.54, and − 0.58 in the placebo, finasteride, doxazosin, and combination groups at 1 yr. Reductions with doxazosin and combination therapy, but not finasteride, were greater than with placebo at 1 and 4 yr. (Johnson et al. 2007). This study [39] showed no benefit of the use of combination therapy of finasteride and α-AR blockers in treating nocturia over the use α-AR blockers alone. Oelke et al. [40] assessed the impact of dutasteride compared with placebo on nocturia in men with LUTS suggestive of BPH, using pooled data from dutasteride phase III studies. In total, 4321 patients with a mean age of 66 years were evaluated using Question 7 of the IPSS questionnaire. They found that from month 12 onwards, mean nocturia improvements were significantly superior with dutasteride than with placebo. These results were confirmed after 24 months of treatment. In another study, Oelke et al. [41] used data from the COMBAT study to assess the impact of dutasteride plus tamsulosin combination therapy, compared with dutasteride or tamsulosin monotherapy, on nocturia in men with LUTS/BPH). Nocturia was assessed using Question 7 of the IPSS questionnaire. In total, 4722 patients with a mean age of 66 years were included. The authors found that nocturia improvements were significantly better with combination therapy than with either monotherapy, and concluded that their analyses were the first to show greater improvement with a 5α-reductase inhibitors/α_1_-AR blocker combination versus either agent alone for the management of nocturia in patients with LUTS/BPH. Studies specifically designed to assess nocturia are required to prospectively confirm these effects of 5α-reductase inhibitors.

### Antimuscarinics

Antimuscarinics are still the gold standard for treatment of the OAB syndrome which includes nocturia. The rationale for using antimuscarinics in OAB has been discussed elsewhere and is based on reducing the generation of bladder afferent activity and frequency of voiding [10]. The reasons why antimuscarinics should have an effect on nocturnal polyuria are unclear, and significant effects have not been convincingly demonstrated in clinical studies. An effect in nocturia patients suffering from nocturnal urgency would expected, and some studies demonstrated diminished urgency-related nocturnal voids [42, 43], but none of the antimuscarinics studied decreased nocturic episodes. Even patients with diminished bladder capacity due to OAB seem to have shown little clinical improvement in nocturia patients. In a study of 962 men and women with OAB-related nocturia, 10 mg of solifenacin caused a 0.12 decrease in nocturnal voids [44]. A study of tolterodine extended release (Tolt ER) saw no significant decrease in the number of nocturic episodes in 850 patients with OAB-related nocturia [42]. In a study of 658 patients, those treated with trospium chloride decreased nocturic episodes by 0.57 per night, while those receiving placebo improved their nocturia by 0.29 episodes [45]. Several studies have shown fesoterodine to have no effect on reducing nocturic episodes in patients with OAB despite a significant reduction in the number of diurnal voids [46–48]. However, it cannot be excluded that some patients with nocturnal urgency may benefit from antimuscarinic treatment.

### Various Agents

#### Melatonin

Drake et al. [49] investigated melatonin as a potential treatment for nocturia associated with bladder outflow obstruction in older men. A total of 20 men with urodynamically confirmed bladder outflow obstruction and nocturia were entered into a randomized, double-blind, placebo-controlled crossover study assessing the effect of 2 mg controlled release melatonin at night on nocturia. Melatonin and placebo caused a decrease in nocturia of 0.32 and 0.05 episodes/night (*p* = 0.07). Nocturia responder rates (a mean reduction of at least − 0.5 episodes/night) were higher with active medication (*p* = 0.04). Daytime urinary frequency and IPSS were minimally altered. The adverse effect profile was good, but the authors questioned whether the found differences were clinically significant.

Sugaya et al. [50] compared the effects of melatonin and the hypnotic, rilmazafone hydrochloride, in elderly patients with nocturia. The patients were randomly divided into two groups: one group received melatonin (2 mg/day) and the other group received rilmazafone hydrochloride (2 mg/day) for 4 weeks. A total of 42 patients (25 men and 17 women, aged 65–79 years) were enrolled. In both the melatonin- and rilmazafone-treated groups, the mean number of nocturnal urinations was significantly decreased after 4 weeks compared with baseline. However, the mean number of daytime urinations was unchanged.

A protocol to evaluate whether melatonin reduces the frequency of nocturia episodes in MS patients has been published, but no results are available [51].

#### Mirabegron

Like for antimuscarinics, there is no convincing rationale for mirabegron having an effect on nocturnal polyuria. Since mirabegron has documented efficacy and has been approved for the treatment of the OAB syndrome, some effect on nocturnal voiding frequency might be expected. Data from a phase 2 dose-ranging study of mirabegron in a mixed population with OAB, where nocturia was evaluated as a secondary parameter, showed that mirabegron 50 mg reduced significantly the number of nocturia episodes by 0.6 from baseline vs 0.22 on placebo [52]. Studies specifically designed to assess nocturia are required to prospectively confirm these effects. Whether mirabegron is more effective than antimuscarinics also remains to be established.

#### Tadalafil

Oelke et al. [53] integrated data from four randomized, placebo-controlled, double-blind, 12-week registrational studies of tadalafil for LUTS/BPH, and assessed nocturia as nighttime voiding frequency using the International Prostate Symptom Score question 7 (IPSS Q7). For patients receiving placebo and tadalafil, respectively, the proportion with improved nocturnal frequency was 41.3 and 47.5%, with no change 44.8 and 41.0%, and with worsening was 13.9 and 11.5%. Even if the results showed a statistically significant improvement in nocturnal frequency with tadalafil over placebo, the treatment difference was small and not considered clinically meaningful.

#### Imipramine

The tricyclic antidepressant, imipramine, is an approved treatment for nocturnal enuresis in children, but use of this drug has not been studied in adults with nocturia. Adverse effects include cardiac arrhythmias, hepatotoxicity, CNS depression, drug interactions, and possible overdose. Thus, imipramine should not be considered standard therapy for nocturia in adults [2].

### Combinations

Several studies have shown advantages with combination treatment. Many combinations have been used, some of which are exemplified below.

Fu et al. [24] studied the effects of combined desmopressin and furosemide in a total of 122 patients who had a mean of two or more voids per night. A 3-week dose-titration phase established the optimum desmopressin dose (0.1, 0.2, or 0.4 mg). After a 1-week “washout” period, patients who showed sufficient response during the dose-titration period were randomized to receive furosemide (20 mg, 6 h before bedtime) and the optimal dose of desmopressin or placebo (at bedtime) in a double-blind design for 3 weeks. In total, 82 patients were randomized into the double-blind treatment period and 80 (58 men and 22 women, mean age 67 years, SD 8 years) completed the study. Compared to placebo, furosemide and desmopressin combined resulted in a significant reduction in the mean number of nocturnal voids (43 vs. 9%), nocturnal urine volume (37 vs. 5%), and increase in the mean duration of the first sleep period (52 vs. 19%).

In a double-blind, randomized, proof-of-concept study, Rovner et al. [54•] enrolled female 106 patients (≥ 18 years), with overactive bladder (OAB) and nocturia, with ≥ 2 nocturnal voids, receiving a 3-month once-daily combination (desmopressin 25 μg, orally disintegrating tablets [ODT]/tolterodine 4 mg [Detrol® LA]; *n* = 49) or monotherapy (tolterodine 4 mg/placebo ODT; *n* = 57). Post-hoc exploratory analysis was performed for patients with and without baseline nocturnal polyuria (*n* = 47 each). In post-hoc analysis, nocturnal polyuria patients showed a benefit with combination versus monotherapy for nocturnal void volume (*p* = 0.034) and time to first nocturnal void (*p* = 0.045), and a non-significant improvement in Nocturia Impact Diary scores. It was concluded that low-dose desmopressin could be safely combined with tolterodine for treating nocturia in women with OAB, with a significant benefit in women with nocturnal polyuria.

In men 40 to 65 years old with lower urinary tract symptoms and persistent nocturia despite α-blocker therapy for at least 8 weeks, Kim et al. [55•] studied the effect of add-on desmopressin. Patients were randomized to once daily placebo or desmopressin 0.2 mg for 8 weeks. A total of 86 patients were randomized to treatment (placebo *n* = 39; desmopressin *n* = 47). The desmopressin add-on group was significantly superior to placebo in terms of the change from baseline in the mean number of nocturia episodes, the changes in nocturnal urine volume, total International Prostate Symptom Score, the nocturnal polyuria index, the ICIQ-N (International Consultation on Incontinence Questionnaire-Nocturia), and the willingness to continue. Adverse events were similar in the two groups.

## Summary and Conclusions

Nocturia is a common complaint with a significant effect on quality of life. The pathophysiology of the disorder is often multifactorial, but two main mechanisms have been identified, occurring alone or in combination: low functional bladder capacity and nocturnal polyuria. The multifactorial pathophysiology not only implies several possible targets for therapeutic intervention but also means that it is unlikely that one treatment modality including drugs will be successful in all patients. Several pharmacological principles have been tested with varying success. The treatment of choice should depend upon the main underlying cause, thus aiming primarily to increase bladder capacity by counteracting detrusor overactivity and/or reducing nocturnal polyuria. Denys et al. [56] suggested that in studies, patients with nocturia should be treated according to the underlying pathophysiology: (1) antimuscarinics or β3-agonists for OAB symptoms, (2) α_1_-AR blockers or 5α-reductase inhibitors in men with BPO caused by enlarged prostates, (3) desmopressin or diuretics for nocturnal polyuria, (4) continuous positive airway pressure in nocturic patients with obstructive sleep apnoea, and (5) all its combinations in case of combined pathophysiology. Drugs approved for the treatment of male LUTS and OAB are known to be far more effective for treatment of the daytime symptoms than for nocturia. Desmopressin is the only therapeutic agent to be highly recommended for treating nocturnal polyuria by current guidelines. There is a medical need for new, approved drugs for treatments for patients with nocturia. However, using current available agents, effective, personalized treatment may be designed taking into account gender, co-morbidities, and identified etiological factors.
